# LRRC75A-AS1 facilitates breast cancer cell proliferation and invasion via functioning as a CeRNA to modulate miR489-3p/ARD1

**DOI:** 10.1038/s41598-025-17372-9

**Published:** 2025-08-26

**Authors:** Chunjiao Yu, Zhiyuan Wang, Xi Zhang, Ming Yu, Xue Cao, Hongbo Zhao, Shan Yan

**Affiliations:** 1 Institute of Biomedical Engineering, Kunming Medical University, 1168 West Chunrong Road, Chenggong District, Kunming, 650500 Yunnan P.R. China; 2 Yunnan Key Laboratory of Breast Cancer Precision Medicine, Chenggong District, Kunming, Yunnan People’s Republic of China; 3 Department of Pathology, The First Affiliated Hospital of Kunming Medical University, Kunming, Kunming, 650031 Yunnan P.R. China; 4 Department of Clinical Laboratory, Yunnan Cancer Center), The Third Affiliated Hospital of Kunming Medical University (Yunnan Cancer Hospital, Kunming, 650118 P.R. China; 5 Department of Laboratory Animal Science, Kunming Medical University, Kunming, 650500 P.R. China

**Keywords:** Arrest defective 1, Breast cancer, LRRC75A-AS1, MiR-489-3p, Cancer, Breast cancer

## Abstract

This study aimed to elucidate the molecular mechanism through which ARD1 regulates breast cancer (BC) progression via the LRRC75A-AS1/miR-489-3p axis. The expression levels of ARD1, miR-489-3p, and LRRC75A-AS1 in BC cells were quantified using reverse transcription-polymerase chain reaction (RT-PCR). The interaction between miR-489-3p and ARD1 was validated through dual-luciferase reporter assays and RNA-binding protein immunoprecipitation (RIP). The sponge effect of LRRC75A-AS1 on miR-489-3p was confirmed by RNA pull-down assays. Functional roles of LRRC75A-AS1, miR-489-3p, and ARD1 in cell proliferation, invasion, and epithelial-to-mesenchymal transition (EMT) were evaluated using colony formation, Transwell, and western blot assays. Moreover, in vivo tumor xenograft experiments were conducted in BALB/c nude mice to assess the effect of LRRC75A-AS1 knockdown and its interaction with miR-489-3p and ARD1 on tumor growth. ARD1 promoted BC cell proliferation, invasion, and EMT. miR-489-3p was identified as a negative regulator of ARD1, while LRRC75A-AS1 acted as a competing endogenous RNA (ceRNA) that sponged miR-489-3p, thereby restoring ARD1 expression. Rescue experiments confirmed that LRRC75A-AS1 facilitated BC cell malignancy via the miR-489-3p/ARD1 axis. Importantly, in vivo studies demonstrated that silencing LRRC75A-AS1 significantly inhibited tumor growth in nude mice, accompanied by reduced ARD1 expression and increased miR-489-3p levels. The inhibitory effect on tumor growth was reversed by miR-489-3p inhibition and further restored by ARD1 knockdown, validating the functional relevance of this regulatory axis in vivo. Both in vitro and in vivo findings reveal that LRRC75A-AS1 promotes breast cancer progression by sponging miR-489-3p and upregulating ARD1. The LRRC75A-AS1/miR-489-3p/ARD1 ceRNA axis represents a novel regulatory pathway and a promising therapeutic target in BC.

**Supplementary Information:**

The online version contains supplementary material available at 10.1038/s41598-025-17372-9.

## Background

Breast cancer (BC) has surpassed lung cancer to become the most prevalent cancer among women, representing 2.3 million new cases and approximately 11.7% of all cancers in 2020^1^. Despite significant advancements in early diagnosis and systematic therapeutic approaches, BC continues to be the foremost cause of death in women^2–4^. The exact mechanism of BC development and progression remains elusive. Therefore, gaining insight into the molecular mechanisms underlying BC development and identifying therapeutic targets are crucial for enhancing the prognosis of BC patients.

Arrest Deficient Protein 1 (ARD1) is an acetyltransferase that catalyzes the transfer of acetyl groups from acetyl-CoA, covalently binding them to the α-residue at the N-terminus of the nascent peptide chain^5,6^. ARD1 plays pivotal roles in the cell cycle, proliferation, apoptosis, migration, oxidative stress response, as well as growth and survival. Abnormal levels of ARD1 may lead to biological dysfunction, neurodegeneration, atherosclerosis, and various tumor diseases^7–9^. Our previous studies have indicated that ARD1 is involved in promoting breast cancer; however, its regulatory mechanisms have yet to be fully elucidated.

Long non-coding RNAs (LncRNAs) and microRNAs (miRNAs) regulate diverse physiological processes, including chromatin remodeling, RNA transcription, RNA splicing, and protein translation, among others, which are pivotal from normal life activities to diseases^10,11^. Recently, an innovative mechanism was proposed whereby LncRNAs act as “miRNA sponges” to regulate tumorigenesis and progression by functioning as competitive endogenous RNAs (ceRNAs), preferentially combining with shared sequences of miRNAs to modulate the expression of downstream miRNA target genes^11,12^. Zhang and colleagues found that the LncRNA LINC00963 promotes tumorigenesis and radiosensitivity in breast cancer by competitively binding to miR-324-3p, antagonizing its inhibitory effect on ACK1, thereby increasing AKCI levels^13^. In another study, the LncRNA NONHSAT101069, serving as a ceRNA, was found to regulate breast cancer progression by sequestering miR-129-5p and inducing Twist, the target protein of miR-129-5p^14^. Prior studies have also demonstrated that miRNA miR-489-3p inhibits invasion and metastasis of osteosarcoma cells^15^while LncRNA LRRC75A-AS1 promotes proliferation, metastasis, invasion, and clone formation of colorectal cancer cells^16^yet their roles and regulatory mechanisms in breast cancer remain unknown.

In this study, we elucidated the role of LRRC75A-AS1 and miR-489-3p in breast cancer. Mechanistically, the LncRNA LRRC75A-AS1 acts as a miR-489-3p ceRNA sponge that antagonizes the activity of miR-489-3p on its target protein ARD1, thereby upregulating ARD1 levels and promoting breast cancer progression, thus offering a potential therapeutic target for breast cancer.

## Materials and methods

### Tissue sample

Twenty-two pairs of cancer and para-carcinoma tissue samples were obtained from breast cancer patients who had not undergone radiotherapy and chemotherapy before surgery at Yunnan Cancer Hospital. The research protocol was approved by the Ethics Committee of Kunming Medical University, and all subjects provided informed consent. The collected tissue samples were stored in RNA later at −80 °C.

## Cell culture

The MDA-MB-231 (gift from Prof. Ceshi Chen, Institute of Zoology in Kunming, Chinese Academy of Sciences) was used in this study, and cultured in DMEM/F12 medium. SK-BR-3 was purchased from Nanjing Dongji Corporation and cultured in DMEM medium. The medium contained 10% fetal bovine serum, 100 M/mL penicillin, and 100 µg/mL streptomycin. Breast cancer cells were cultured in a cell culture incubator with 5% CO2 at 37 °C.

## Cell transfection

First, cells were evenly seeded into six-well plates overnight. Then, 1 µg of plasmid or 10 µL of mimic fragment and 3 µL of Lipofectamine 3000 were diluted with 50 µL of serum-free medium Opti-MEM, respectively. Subsequently, mix them gently and incubate at room temperature for 20 min. Next, put the mixed solution into a 6-well plate with the cells to be transfected. Lastly, replace the complete medium after 6 h of incubation and culture further for 48 h.

## Quantitative RT-PCR

Total RNA was extracted using Trizol reagents (Invitrogen, Carlsbad, CA) according to the supplier’s protocol. For lncRNAs or miRNAs, reverse transcription reactions were performed using SuperScript™ II reverse transcriptase (Invitrogen, Carlsbad, CA) for lncRNAs and miScript reverse transcription kit (Qiagen, Hilden, Germany) for miRNAs. qRT-PCR was conducted using SYBR@Premix Ex TaqTM (TaKaRa Bio Group, Shiga, Japan). Relative normalization of GAPDH or U6 expression was assessed by 2-ΔΔCt.

## MTT assay

The MTT solution was purchased from Promega for the cell proliferation assay. After 48 h of transfection, breast cancer cells were seeded into 96-well plates at a concentration of 5000 cells per well, followed by the addition of 20 µL MTT solution for 2 h in 5% CO2 at 37 °C. Cell proliferation was assessed at the indicated times by measuring the absorbance at 490 nm using a spectrophotometer.

### Transwell assay

Transwell plates (Corning, NY, USA) pre-coated with Matrigel (Corning, NY, USA) were utilized to assess the invasive abilities of cells. The upper layer of the chamber was inoculated with 1 × 104 cells suspended in serum-free medium, while the lower layer contained complete culture medium with 20% FBS. After 24 h, cells that had crossed the membranes were fixed with 4% formaldehyde and stained with crystal violet. Subsequently, cells were observed and imaged under a Nikon microscope. Any remaining cells in the upper chamber were removed with a swab.

## Colony formation assay

After 48 h of transfection, cells were harvested for the colony formation experiment. Cells at a concentration of 5 × 102 per well were seeded in a 6-well plate and cultured at 37 °C for 2 weeks. Upon colony formation, the culture medium was aspirated, and the cells were washed with PBS. Subsequently, they were fixed and stained with 4% paraformaldehyde and 0.4% crystal violet solution (SolarbioChina, Beijing) for 15 min, respectively. Finally, the colony formation ability was assessed by counting the number of stained colonies.

## RNA Immunoprecipitation (RIP)

The Magna RNA Immunoprecipitation (RIP) kit (Millipore, Billerica, USA) was utilized for RIP analysis in MDA-MB-231 and SK-BR-3 cells. Magnetic beads containing Ago2 or IgG (negative control) antibodies were added to the cell lysate previously stored in the RIP buffer. Purified RNA was examined by qRT-PCR to detect the relative expression of LRRC75A-AS1, miR-489-3p, and ARD1.

### Luciferase reporter assay

Construct LRRC75A-WT (or ARD1-WT) and LRRC75A-Mut (or ARD1-Mut) into the pGL3-basic plasmid. The constructed plasmid was co-transfected with miR-489-3p mimics or NC mimics into MDA-MB-231 and SK-BR-3 cells for 48 h. The Dual-Luciferase^®^ Reporter Assay System (Promega, E1910) was utilized to measure relative luciferase activity.

### Western blot analysis

MDA-MB-231 and SK-BR-3 cells were lysed using RIPA lysis buffer (Solarbio). Primary antibodies, including E-cadherin (ab76055), N-cadherin (ab18203), Vimentin (ab45939), and ARD1 (ab155687), were purchased from Abcam (Cambridge, UK). GAPDH was detected using an ECL detection reagent (Tanon) from Zhengergic Biological (Chengdu).

### FISH assay

The RiboTM Fluorescent In Situ Hybridization Kit (Ribobio, C10910) was employed to conduct RNA FISH assays for observing the subcellular localization of LRRC75A-AS1 in MDA-MB-231 and SK-BR-3 cells (6 × 104). The cells were fixed with 4% paraformaldehyde at room temperature for 10 min. Then, 0.5% Triton X-100 was used to permeabilize the fixed cells. Following that, RiboTM h-18 S FISH Probe Mix (Red) (Ribobio, lnc110102) was used to incubate the cells overnight at 37 °C. DAPI was utilized to stain the cell nuclei at room temperature for 10 min. The anti-fluorescence quenching solution was added and placed at 4 °C away from light or photographed directly under a confocal laser fluorescence microscope.

### Animal experiments

All animal experiments were approved by the Animal Ethics Committee of Kunming Medical University. For the in vivo tumor growth assay, four-week-old BALB/c nude mice were randomly divided into four groups (*n* = 8 per group). To establish the xenograft model, MDA-MB-231 cells (5 × 10^6 cells in 100 µL PBS) stably transfected with control vector, sh-LRRC75A-AS1, sh-LRRC75A-AS1 + miR-489-3p inhibitor, or sh-LRRC75A-AS1 + miR-489-3p inhibitor + sh-ARD1 were subcutaneously injected into the right flanks of mice. Tumor size was measured every 3 days using a caliper, and tumor volume was calculated using the formula: Volume = (length × width²)/2. After 22 days, mice were euthanized, and tumors were harvested, weighed, and processed for histological and molecular analysis.

### Hematoxylin and Eosin (H&E) staining

Tumor tissues were fixed in 4% paraformaldehyde, embedded in paraffin, and sectioned at a thickness of 4 μm. Sections were deparaffinized in xylene and rehydrated through a graded ethanol series. Standard hematoxylin and eosin staining was performed to observe histological changes. Slides were examined under a light microscope at 100× and 200× magnifications.

### Immunohistochemistry (IHC)

Paraffin-embedded tumor sections were deparaffinized and rehydrated, followed by antigen retrieval in citrate buffer (pH 6.0) using microwave heating. Endogenous peroxidase activity was blocked with 3% hydrogen peroxide for 10 min at room temperature. After blocking with 5% BSA, sections were incubated overnight at 4 °C with primary antibodies against Ki67 (ab115580), ARD1, E-cadherin, N-cadherin, and vimentin. After washing, sections were incubated with HRP-conjugated secondary antibodies for 30 min at room temperature, followed by DAB chromogenic detection and hematoxylin counterstaining. Images were captured using a light microscope (100× or 200×).

### Statistical analysis

GraphPad Prism 6.0 software was utilized to compile and analyze data. Measurement data were described by mean ± standard deviation or median (interquartile range). Comparison between groups was determined by an independent samples t-test or Mann-Whitney U test. Repeated-measures analysis of variance was used for repeated-measures data. The chi-square test or Fisher’s exact test was applied for count data. *P* < 0.05 was considered statistically significant.

#### Ethical approval and consent to participate

The study was conducted in accordance with the Declaration of Helsinki and was approved by the agency Review Committee of Medical Ethics Committee of the Second Affiliated Hospital of Kunming Medical University. Obtain written informed consent from all patients and/or their legal guardians.

## Results

### ARD1 regulates the proliferation and metastasis of BC cells

**Fig. 1:**
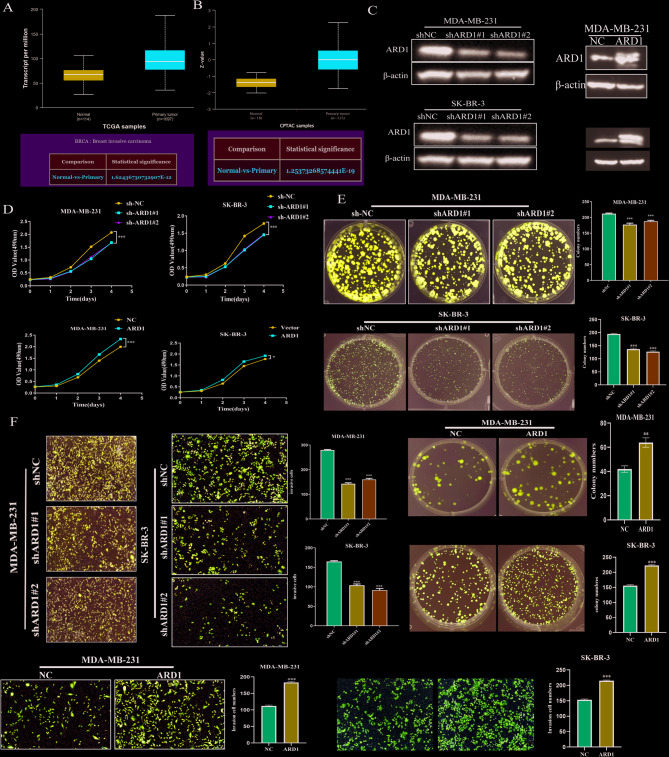
ARD1 regulated BC cell proliferation and metastasis. (**A**, **B**) ARD1 expression levels in breast cancer and normal tissues based on TCGA and CPTAC datasets. (**C**) Western blot analysis of ARD1 knockdown efficiency in MDA-MB-231 and SK-BR-3 cells and overexpression in MDA-MB-231 cells. (D) MTT assays showing cell proliferation after ARD1 knockdown or overexpression. (**E**) Colony formation assays demonstrating the effects of ARD1 on clonogenicity. (**F**) Transwell assays assessing migration and invasion capabilities following ARD1 knockdown or overexpression. Data are presented as mean ± SD. **P < 0.01, ***P < 0.001 versus control.

We analyzed ARD1 levels using TCGA data (which includes 114 normal tissues and 1097 breast cancer tissues) and found that ARD1 expression was significantly higher in primary tumor samples than in normal tissues (Fig. 1a). Exploration in the CPTAC database demonstrated a similar trend (Fig. 1b). Expression and prognosis of ARD1 in the breast cancer patients were also analyzed in the Breast Cancer Gene-Expression Miner (http://bcgenex.ico.unicancer.fr/BC-GEM/GEM-Accueil.php?js=1). The results showed that ARD1 in the tumor tissues were higher than the adjacent tissues (Fig. S1a). The disease-free survival (DFS), overall survival (OS), and distant metastasis-free survival (DMFS) of the high ARD1 expression of breast cancer were all lower in the high ARD1 expression breast cancer, whether in the microarray data (Fig. S1b, c, d) or in the RNA-seq data (Fig. S1e, f) (the website does not contain prognosis data of DMFS in RNA-seq), suggesting the poor prognosis of high ARD1 expression in breast cancer. Western blot was employed to detect the downregulation efficiency of shARD1#1/2 transfected MDA-MB-231 cells. As expected, shARD1#1/2 significantly reduced ARD1 expression (Fig. 1c). Next, a series of functional analyses were conducted. We observed that silencing of ARD1 dramatically reduced cell viability (Fig. 1d). Moreover, clonogenicity was also reduced in ARD1 knockdown cells (Fig. 1e). Wound healing and Transwell assays further demonstrated that silencing ARD1 suppressed the metastatic ability in BC cells (Fig. 1f, g). In summary, ARD1 promotes BC cell proliferation and metastasis.

### MiR-489–3p targeted ARD1 and negatively regulated ARD1 expression

**Fig. 2:**
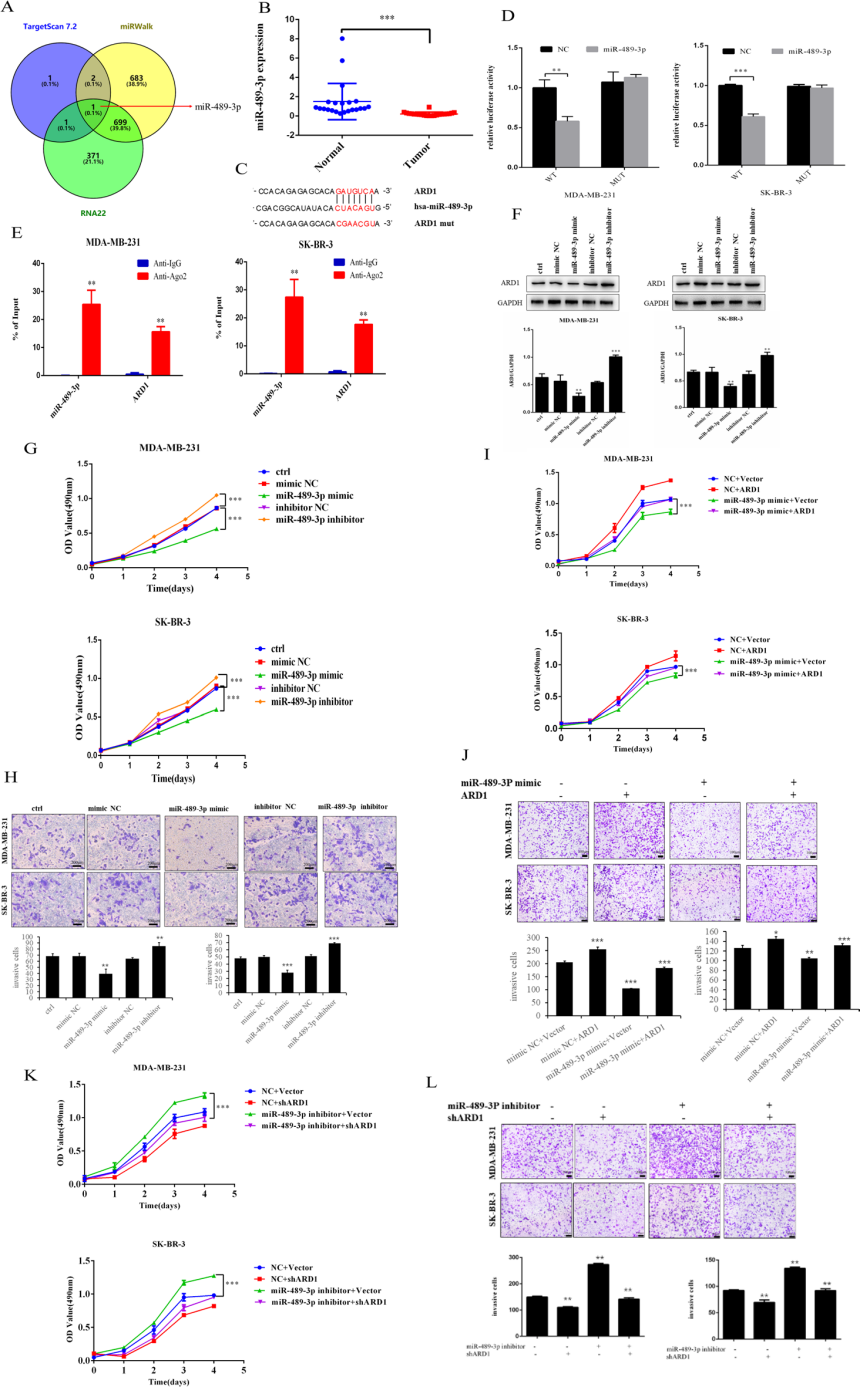
MiR-489–3p targeted ARD1 and negatively regulated ARD1 expression. (**A**) ARD1-associated miRNAs predicted by miRWalk, RNA22, and TargetScan 7.2. (**B**) Expression levels of miR-489-3p in 22 cases of breast cancer and adjacent normal tissues. (**C**) Predicted binding sites of miR-489-3p in ARD1 3’ UTR and its mutation sites. (**D**) Luciferase assay was performed in wild-type or mutant ARD1 and miR-489-3p or NC co-transfected breast cancer cells. (**E**) RIP assay detected the enrichment of miR-489–3p and ARD1 in anti-Ago2 and anti-IgG groups. (**F**) Western blotting analysis of the effect of miR-489-3p on ARD1 protein expression in breast cancer cells.(**G**-**L**) The proliferation and invasive capabilities of MDA-MB-231 and SK-BR-3 cells was detected by MTT and transwell assays. *, *p* < 0.05; **, *p* < 0.01, ***, *p* < 0.001.

Since miRNAs have been reported to target mRNAs and regulate their expression in the progression of many diseases, we searched the database for miRNAs that bind to ARD1 mRNA. Using miRWalk, RNA22, and TargetScan7.2 databases to predict the intersection with Venny2.1, the results suggested that miR-489-3p was a miRNA binding target of ARD1 mRNA (Fig. 2a). We detected the expression of miR-489-3p in the tissues of 22 cases of breast adenocarcinoma and adjacent tissues by qRT-PCR, revealing that the expression of miR-489-3p in breast cancer was significantly lower than that in adjacent normal tissues (Fig. 2b).

Subsequently, the overexpression or knockdown efficiency of miR-489–3p was verified by qRT-PCR in cells transfected with miR-489-3p-mimics or miR-489-3p-inhibitor (Fig. S2a). Figure 2c revealed the binding site between wild-type/mutant ARD1 and miR-489-3p. A luciferase reporter experiment was conducted to verify the validity of the indicated binding sites. Overexpression of miR-489-3p decreased the luciferase activity of wild-type ARD1, but did not affect the mutant luciferase activity (Fig. 2d). RIP analysis showed that the binding of ARD1 mRNA and miR-489-3p to AGO2 was increased (Fig. 2e). Additionally, the regulatory effect of miR-489-3p on ARD1 expression was confirmed. The results revealed that inhibition of miR-489-3p significantly up-regulated ARD1 expression in BC cells (Fig. 2f, S2b), while overexpression of miR-489-3p significantly down-regulated ARD1 expression in both cells. In conclusion, miR-489-3p can bind to ARD1 and negatively regulate its expression in BC cells.

Transfection with miR-489-3p mimic reduced the proliferation and invasion of breast cancer cells, while transfection with miR-489-3p inhibitor increased the proliferation and invasion of breast cancer cells (Fig. 2g, h). Additionally, we demonstrated that the upregulation of migration and invasion could be reversed in the overexpressed BC cell line using miR-489-3p mimics (Fig. 2i, j). Furthermore, the miR-489-3p inhibitor could reverse the downregulated migration and invasion capacity in the knocked down BC cell line (Fig. 2k, l). These results indicated that miR-489-3p inhibited the proliferation and invasion of breast cancer cells by negatively regulating ARD1.

### LRRC75A-AS1 sponges miR-489–3p and negatively modulates miR-489–3p expression

**Fig. 3:**
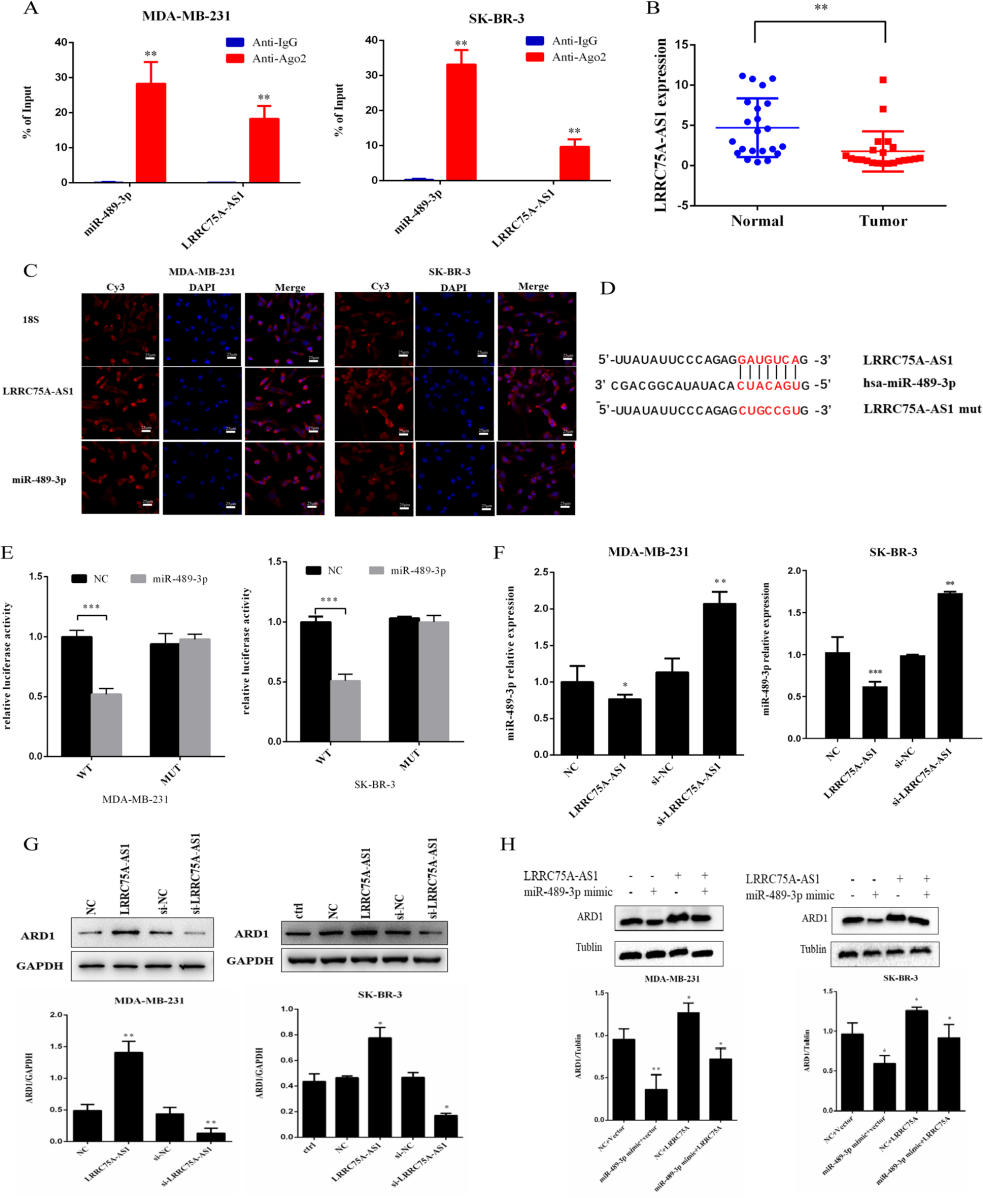
LRRC75A-AS1 negatively regulates miR-489-3p and promotes ARD1 expression through sponge. (**A**) AGO2 RIP was performed in breast cancer cells, and the expression of miR-489-3p and LRRC75A-AS1 associated with AGO2 was detected by qPCR. (**B**) Expression levels of LRRC75A-AS1 in 22 cases of breast cancer and adjacent normal tissues. (**C**) Localization of LRRC75A-AS1 and miR-489-3p in MDA-MB-231 and SK-BR-3 cells was determined by FISH analysis. (**D**) Predicted binding sites and mutations of miR-489-3p in LRRC75A-AS1. (**E**) Luciferase assay was performed in wild-type or mutant LRRC75A-AS1 and miR-489-3p co-transfected breast cancer cells. (**F**) Effect of LRRC75A-AS1 on miR-489-3p expression in breast cancer cells by qPCR. (**G**) Western blot analysis of the effect of LRRC75A-AS1 on the expression of ARD1 protein in breast cancer cells. (**H**) Western blot analysis of the effect of co-transfection of LRRC75A-AS1 and miR-489-3p on the expression of ARD1 in breast cancer cells. *, *p* < 0.05; **, *p* < 0.01, ***, *p* < 0.001.

After confirming that miR-489-3p inhibits ARD1 mRNA, we preliminarily determined that LRRC75A-AS1 binds to miR-489-3p through ENCORI and LncBase database predictions and selected LRRC75A-AS1 as our target for further research. The enrichment of miR-489-3p and LRRC75A-AS1 in the anti-AGO2 group was detected by RIP method (Fig. 3a). Additionally, the expression of LRRC75A-AS1 was significantly higher in BC tissues than in non-tumor tissues (Fig. 3b). The localization of LRRC75A-AS1 and miR-489-3p in MDA-MB-231 and SK-BR-3 cells was further analyzed by FISH, confirming their mainly cytoplasmic localization (Fig. 3c). Therefore, we hypothesize that LRRC75A-AS1 may act as a sponge for miRNAs, preventing them from binding to their target mRNA.

Then, the binding sites between the wild type/mutant LRRC75A-AS1 and miR-489-3p were predicted using Starbase (Fig. 3d). The luciferase reporter experiment showed that the overexpression of miR-489-3p reduced the luciferase activity of the LRRC75A-AS1-WT vector but did not affect the luciferase activity of the mutant vector (Fig. 3e). Thus far, LRRC75A-AS1 has been confirmed as a miR-489-3p sponge in BC.

In addition, si-LRRC75A-AS1 was introduced into BC cells to measure the gene knockout efficiency of LRRC75A-AS1. LRRC75A-AS1 was overexpressed in BC cells, and its overexpression efficiency was detected (Fig. S2c). It was shown that knockdown of LRRC75A-AS1 notably enhanced the expression of miR-489-3p and decreased that of ARD1 at both mRNA and protein levels. Overexpression of LRRC75A-AS1 significantly reduced the expression of miR-489-3p and enhanced the expression of ARD1 at the mRNA and protein levels (Fig. 3f, g, S2d). Moreover, co-transfection of the miR-489-3p mimic rescued the promoted ARD1 expression in LRRC75A-AS1 transfected cells (Fig. 3h). These results indicate that LRRC75A-AS1 promotes the expression of ARD1 by negatively regulating miR-489-3p.

### LRRC75A-AS1 promotes proliferation and invasion of breast cancer cells through the miR-489-3p/ARD1 axis

**Fig. 4:**
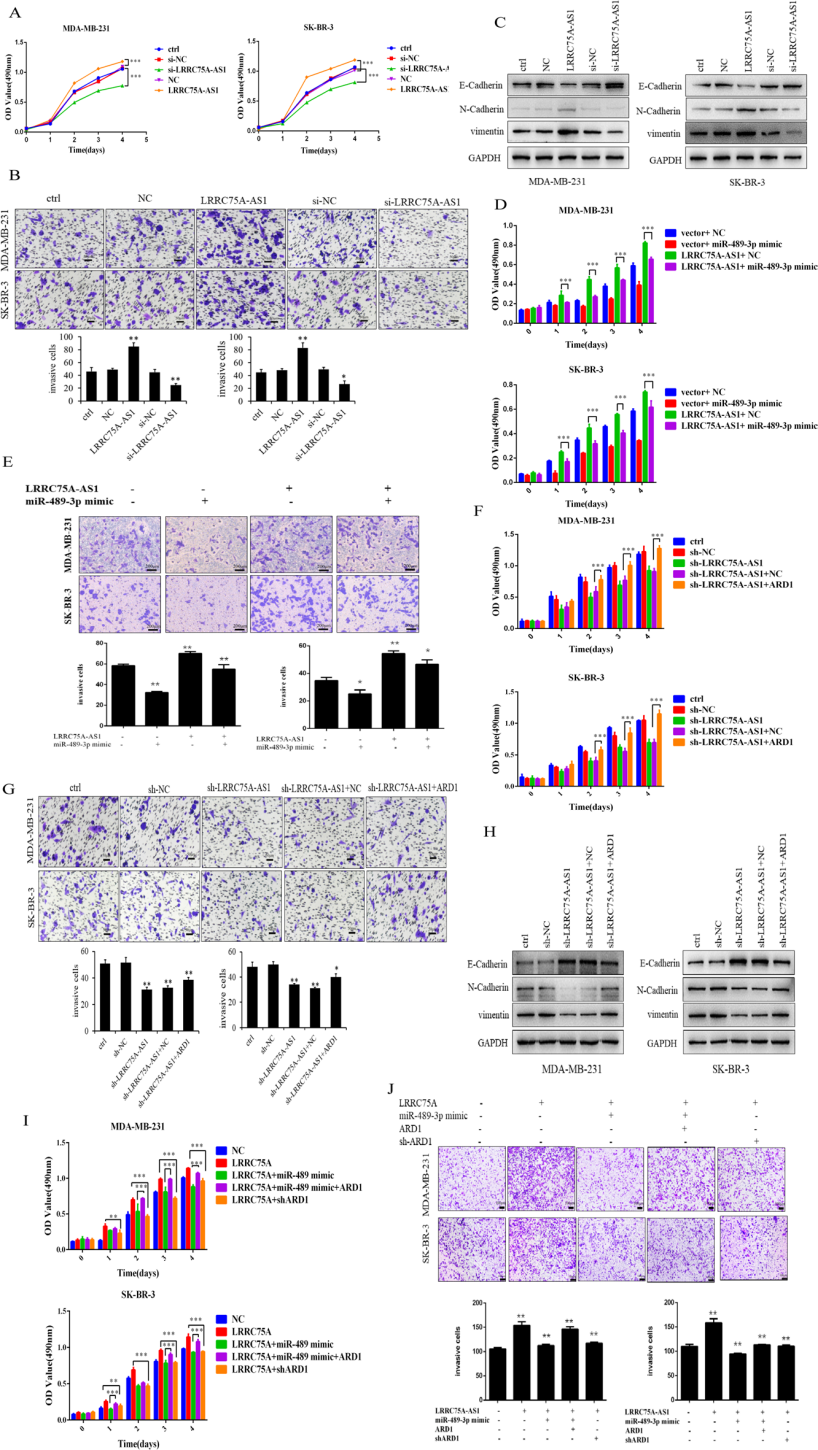
LRRC75A-AS1 promotes the proliferation and invasion of breast cancer cells through the miR-489-3p/ARD1 axis. (**A**) The effects of LRRC75A-AS1 overexpression and knockdown on breast cancer cell proliferation were detected by MTT assay. (**B**) Transwell analysis of the effects of LRRC75A-AS1 overexpression and knockdown on breast cancer cell invasion. (**C**) After knockdown and overexpression of LRRC75A-AS1, EMT-associated proteins were detected by Western blot in MDA-MB-231 and SK-BR-3 cells. (**D**) The effect of co-transfection of LRRC75A-AS1 and miR-489-3p on cell proliferation was detected by MTT assay. (E) Transwell analysis of the invasion capacity of breast cancer cells co-transfected with LRRC75A-AS1 and miR-489-3p. (**F**) The effect of sh-LRRC75A-AS1 and ARD1 co-transfection on cell proliferation was detected by MTT assay. (**G**) Transwell analysis of the invasion capacity of breast cancer cells co-transfected with ARD1 and shLRRC75A-AS1. (**H**) Western blot was used to detect EMT-associated proteins in breast cancer cells co-transfected with ARD1 and shLRRC75A-AS1. (**I**) The MTT assay was used to detect the effect of LRRC75A-AS1, miR-489-3p, and ARD1 co-transfection on cell proliferation. (**J**) Transwell analysis of the invasion capacity of breast cancer cells co-transfected with LRRC75A-AS1, miR-489-3p, and ARD1. *, *p* < 0.05; **, *p* < 0.01, ***, *p* < 0.001.

To verify the function of the LRRC75A-AS1/miR-489-3p/ARD1 network in BC, we conducted a rescue experiment, MTT, Transwell test, and Western blotting, demonstrating that overexpression of LRRC75A-AS1 promoted the proliferation, invasion, and metastasis of BC cells; conversely, LRRC75A-AS1-induced proliferation and invasion were inhibited when miR-489-3p was overexpressed. Knockout of LRRC75A-AS1 undermined the proliferation, invasion, and metastasis ability of BC cells, and overexpression of ARD1 reversed the effects of LRRC75A-AS1 knockdown (Fig. 4a-h).

Overexpression of LRRC75A-AS1 enhanced the proliferation and invasion ability of breast cancer cells. The proliferation and invasion ability of cells overexpressing miR-489-3p was reversed by LRRC75A-AS1 overexpression, while overexpression of ARD1 restored the proliferation and invasion of cells inhibited by miR-489-3p (Fig. 4i, j). These results indicate that LRRC75A-AS1 promotes cell proliferation and invasion through the miR-489-3p/ARD1 axis.

### LRRC75A-AS1 promotes tumor growth and metastasis in vivo by functioning as a sponge for miR-489-3p

**Fig. 5:**
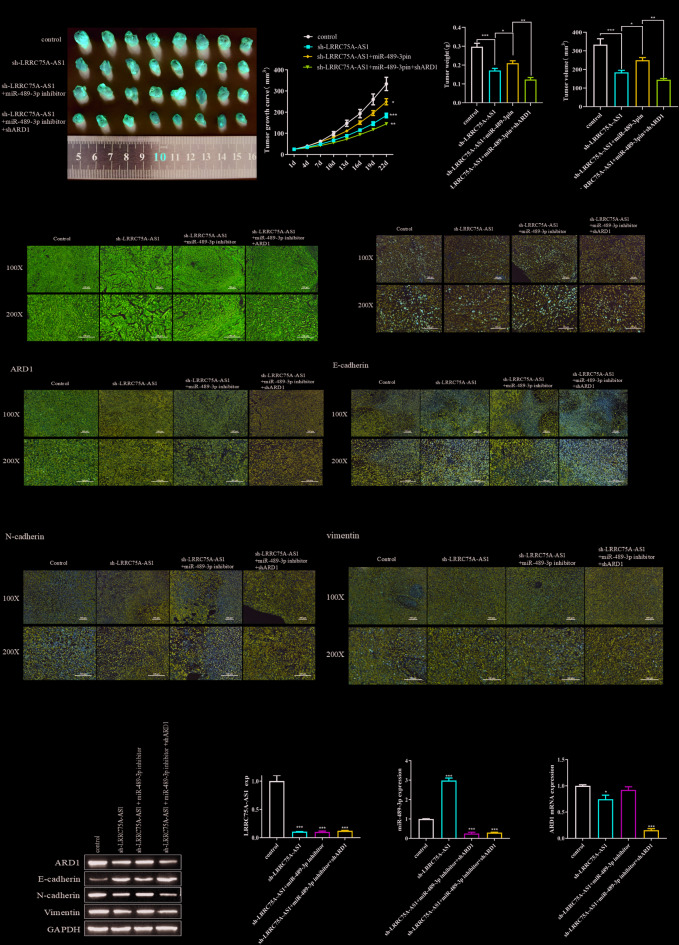
LRRC75A-AS1 promotes breast cancer tumor growth and metastasis in vivo by sponging miR-489-3p and upregulating ARD1 expression. (**A**) Representative images of tumor xenografts from nude mice injected subcutaneously with MDA-MB-231 cells stably transfected with control vector, sh-LRRC75A-AS1, or in combination with miR-489-3p inhibitor and/or ARD1 shRNA. (**B**) Tumor growth curves (left) and quantitative analyses of tumor weight (middle) and tumor volume (right) at endpoint. Data are presented as mean ± SD (*n* = 8 per group). (**C**) HE staining of tumor sections showing changes in tissue structure and cell density across groups (magnifications: 100×, 200×). (**D**) IHC staining for Ki67 to assess tumor cell proliferation in each treatment group (magnifications: 100×, 200×). (**E**) IHC staining for ARD1 and EMT markers (E-cadherin, N-cadherin, vimentin) to evaluate changes in EMT status after each treatment (magnifications: 100×, 200×). (**F**) Western blot analysis of ARD1 and EMT-related protein expression in xenograft tissues. GAPDH served as a loading control. (**G**) qRT-PCR analysis of LRRC75A-AS1, miR-489-3p, and ARD1 mRNA expression levels in tumor tissues from each group. Data are shown as mean ± SD; *P < 0.05, **P < 0.01, ***P < 0.001.

To further elucidate the role of LRRC75A-AS1 in BC progression in vivo, we established a xenograft model by subcutaneously injecting MDA-MB-231 cells stably transfected with either sh-LRRC75A-AS1 or control vector into BALB/c nude mice. Tumor growth curves and tumor weights at endpoint revealed that knockdown of LRRC75A-AS1 significantly suppressed tumor growth compared to the control group (Fig. 5A, B). Notably, co-transfection with a miR-489-3p inhibitor reversed the tumor-suppressive effects of sh-LRRC75A-AS1, supporting a functional interaction between LRRC75A-AS1 and miR-489-3p. Furthermore, silencing ARD1 in this context partially abrogated the miR-489-3p inhibitor-mediated rescue, suggesting that ARD1 acts downstream of the LRRC75A-AS1/miR-489-3p axis.

Histological analysis using H&E staining confirmed reduced tumor cellularity and malignancy in the sh-LRRC75A-AS1 group, which was partially rescued by miR-489-3p inhibition and reversed again upon ARD1 knockdown (Fig. 5C). Immunohistochemical analysis of Ki67 demonstrated significantly decreased proliferative activity in the sh-LRRC75A-AS1 group, consistent with reduced tumor growth, and again, this effect was mitigated by miR-489-3p inhibition and re-suppressed upon ARD1 silencing (Fig. 5D).

Further immunohistochemical evaluation of EMT markers revealed that LRRC75A-AS1 knockdown upregulated E-cadherin and downregulated N-cadherin and vimentin expression, indicative of suppressed EMT. These changes were reversed by miR-489-3p inhibition and re-modulated by ARD1 silencing (Fig. 5E). Western blotting corroborated these findings, showing that LRRC75A-AS1 knockdown reduced ARD1, N-cadherin, and vimentin protein levels while increasing E-cadherin (Fig. 5F).

Finally, qRT-PCR analysis of xenograft tissues demonstrated effective knockdown of LRRC75A-AS1 and ARD1 and confirmed the inverse expression of miR-489-3p in the respective groups (Fig. 5G). Together, these data suggest that LRRC75A-AS1 promotes BC tumor growth and metastasis in vivo by acting as a ceRNA for miR-489-3p, thereby upregulating ARD1 expression and promoting EMT.

## Discussion

**Fig. 6:**
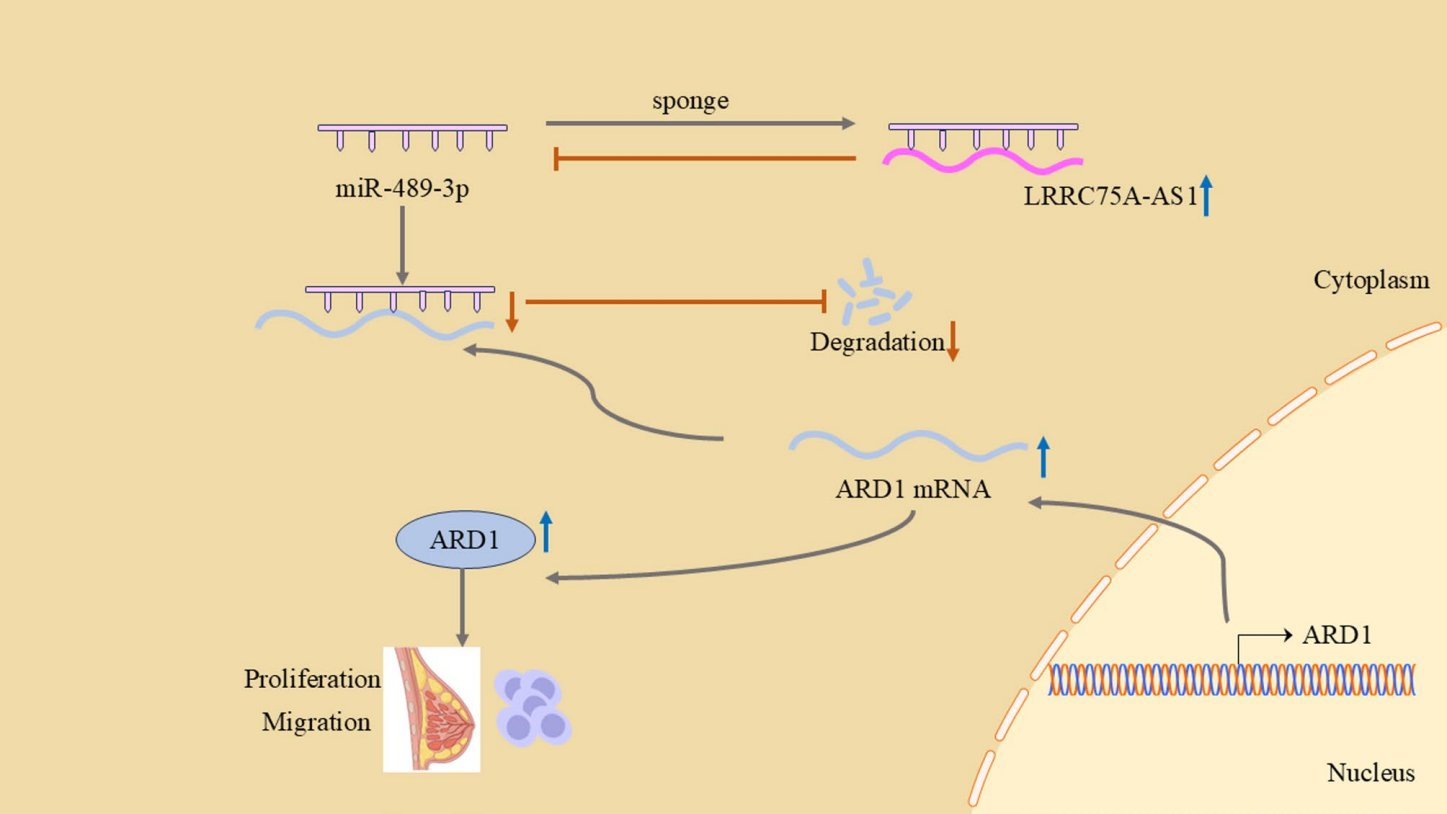
Schematic diagram of LRRC75A-AS1 as a sponge of miR-489-3p competitively binding to miR-489-3p to resist the inhibitory effect of miR-489-3p on ARD1 and thus promote the progression of breast cancer.

Breast cancer is the leading cause of cancer worldwide, and its incidence is expected to increase by 64% from 2011 to 2030^17,18^. However, the pathogenesis of breast cancer remains unclear, involving various gene mutations and a series of complex and diverse biological processes^4,19^. In this study, we identified that the long non-coding RNA LRRC75A-AS1 acts as a competing endogenous RNA (ceRNA) by sponging miR-489-3p, thereby upregulating ARD1 expression and promoting breast cancer progression (Fig. 6).

LncRNAs and miRNAs play pivotal roles in cancer progression^10,11,20^. A novel regulatory mechanism has been proposed: the ceRNA regulatory network describes lncRNAs as miRNA sponges that competitively bind to shared sites of miRNAs and downstream target genes, thereby indirectly regulating the expression of miRNA downstream target genes^21–23^. Cumulative evidence indicates that ceRNA regulatory networks are involved in the biological processes of cancer, including proliferation, invasion, metastasis, EMT, and chemotherapy resistance^24,25^. In our study, we demonstrated that LRRC75A-AS1 acts as a sponge for miR-489-3p, thereby regulating ARD1 expression and promoting breast cancer progression. These findings highlight LRRC75A-AS1 as a potential therapeutic target and diagnostic biomarker for breast cancer.

LRRC75A-AS1 is a small nucleolar RNA host gene whose role in cancer is controversial. Previous studies have reported that LRRC75A-AS1 inhibits the proliferation and migration of colorectal cancer cells, but the specific mechanism is not yet elucidated^16^. In multiple myeloma, LRRC75A-AS1 has been reported to inhibit proliferation and promote apoptosis by modulating the miR-199b-5p/PDCD4 axis^26^. In contrast, Li et al. found that LRRC75A-AS1 functions as a ceRNA that binds to miR-380-3p, positively regulating BAALC expression, and promoting breast cancer cell proliferation, invasion, and EMT^27^which is consistent with our findings. The controversial role of LRRC75A-AS1 may be related to different types of carcinomas.

The role of miR-489-3p is similar to that of LRRC75A- AS1, which plays both tumor-promoting and tumor-suppressive roles. MiR-489-3p promotes cell proliferation, migration, EMT, and tumor formation and metastasis by regulating USP48 and inactivating the Wnt/β-catenin pathway in non-small cell lung cancer^28^. In contrast, miR-489-3p regulates LDHA and PKM2 in pancreatic cancer^29^DLX1 in prostate cancer^30^SIX1 in melanoma^31^and HDAC2 in bladder cancer to inhibit tumor growth^32^. The physiological function of ARD1 involves the cell cycle, oxidative stress, autophagy, development, migration, and others. The role of ARD1 across various tumors has been reported, including breast cancer, liver cancer, osteosarcoma, lung cancer, oral squamous cell carcinoma, and colon cancer, but the roles of ARD1 are also controversial in tumors. Some studies found that high expression of ARD1 is associated with short overall survival (OS) in breast cancer^33^liver cancer^34^lung cancer^35^and osteosarcoma. In contrast, high expression of ARD1 was associated with long overall survival in breast cancer^36,37^lung cancer^38^and squamous cell carcinoma^39^. They play an important role in tumor initiation and development, and their specific functions may be related to the regulation of upstream and downstream molecules in specific environments. This study found that LRRC75A-AS1 negatively regulates miR-489-3p, positively regulates ARD1 levels, and promotes breast cancer progression.

In addition to its direct effects on tumor cell proliferation and invasion, accumulating evidence suggests that lncRNAs and ceRNA networks also contribute to the modulation of the tumor microenvironment (TME). The TME, consisting of extracellular matrix, fibroblasts, immune cells, and endothelial cells, plays a critical role in supporting tumor progression, metastasis, and drug resistance. Recent studies have indicated that lncRNAs can regulate stromal remodeling, immune evasion, and angiogenesis by acting as ceRNAs within the TME. Although our study primarily focused on the tumor cell-intrinsic role of LRRC75A-AS1, it is plausible that LRRC75A-AS1 may also influence components of the TME through miR-489-3p and ARD1 signaling. Further investigations are warranted to explore the potential regulatory effects of LRRC75A-AS1 on immune cell infiltration, cytokine secretion, and stromal cell activation in breast cancer.

Clinically, the identification of the LRRC75A-AS1/miR-489-3p/ARD1 axis provides a promising therapeutic target and potential biomarker for breast cancer diagnosis and treatment. Therapeutic strategies aimed at silencing LRRC75A-AS1 or restoring miR-489-3p levels may offer novel approaches to inhibit tumor growth and metastasis. Additionally, targeting ARD1 directly or modulating this regulatory axis may enhance treatment efficacy, particularly in patients resistant to conventional therapies. Our findings lay the groundwork for further preclinical studies and future clinical translation to evaluate the therapeutic potential of this ceRNA network in breast cancer management.

In conclusion, we identified the lncRNA LRRC75A- AS1 as an oncogene in BC, as it functions as a ceRNA by competitively binding miR-489-3p, inducing ARD1 levels, and promoting breast cancer progression.

## Supplementary Information

Below is the link to the electronic supplementary material.

Supplementary Material 1

Supplementary Material 2

Supplementary Material 3

## Data Availability

All data generated or analysed during this study are included in this published article and its supplementary information files.
