# Supplementary figures and images for: LRRC75A-AS1 facilitates breast cancer cell proliferation and invasion via functioning as a CeRNA to modulate miR489-3p/ARD1
Source: Sci Rep. 2025 Aug 26;15:31501. doi: 10.1038/s41598-025-17372-9 (PMC12381120; doi:10.1038/s41598-025-17372-9)

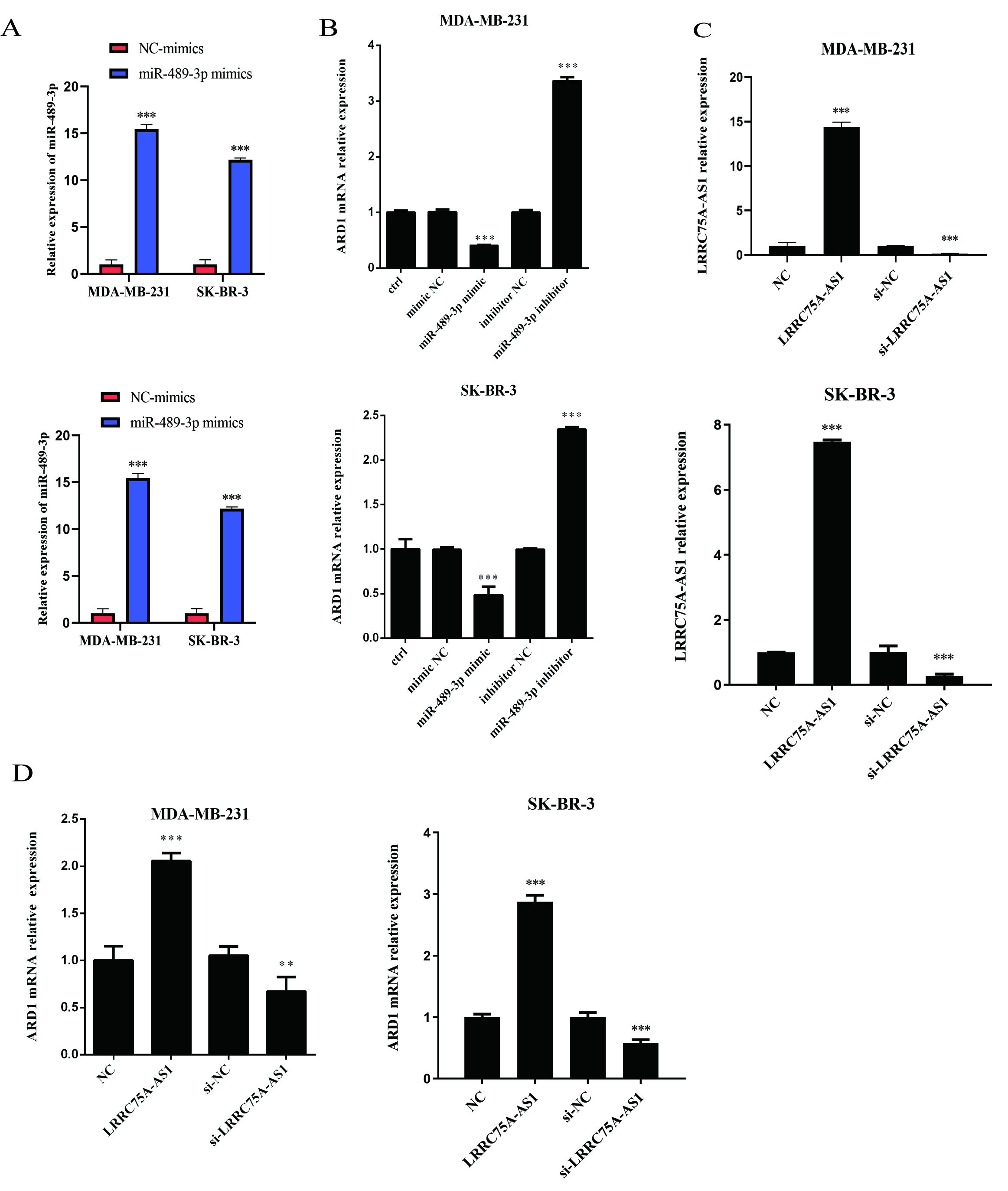

Supplement: Supplementary file 1 — Supplementary Material 1 [file 41598_2025_17372_MOESM1_ESM.tif]

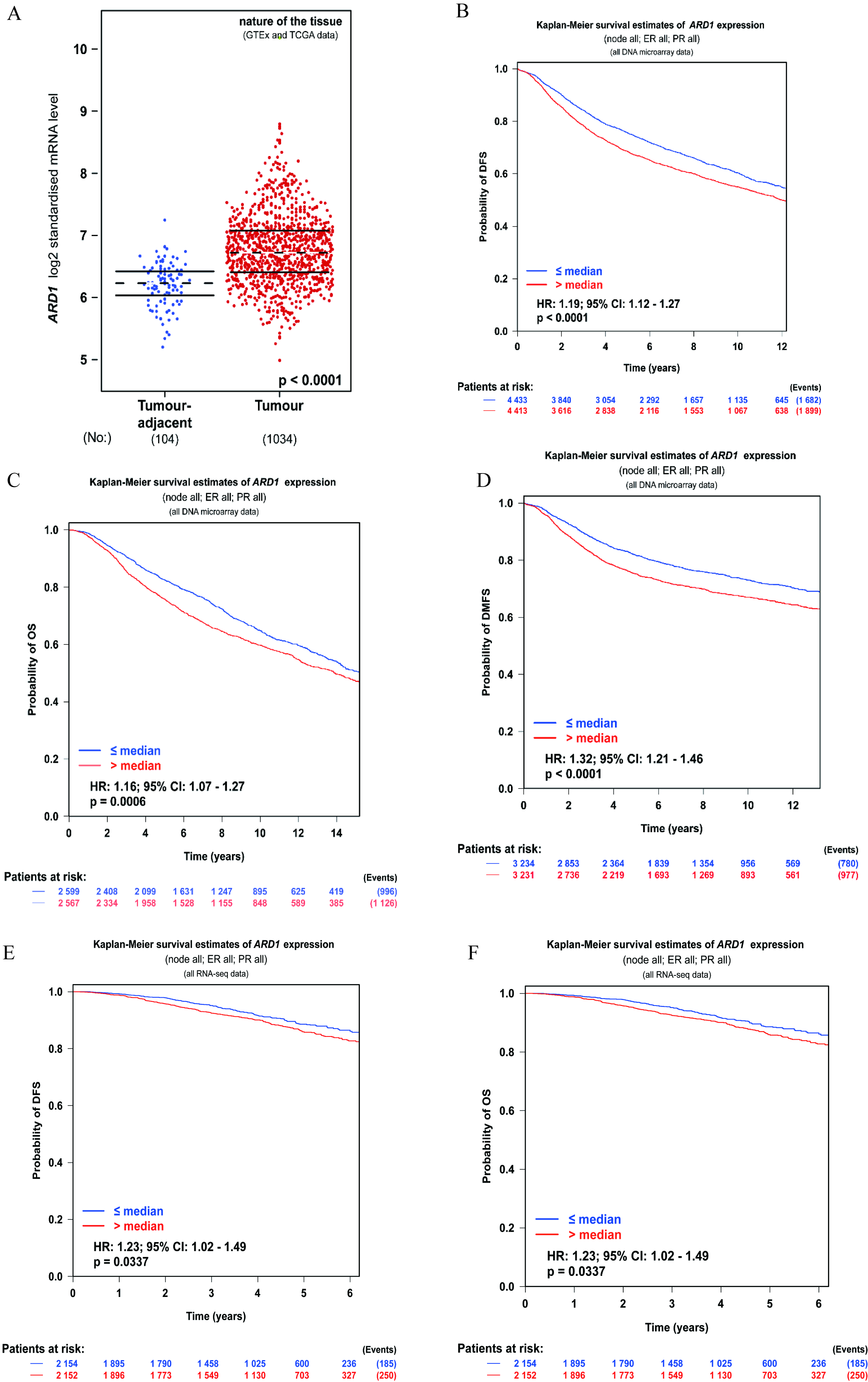

Supplement: Supplementary file 2 — Supplementary Material 2 [file 41598_2025_17372_MOESM2_ESM.tif]
