# Supplementary material for: LRRC75A-AS1 facilitates breast cancer cell proliferation and invasion via functioning as a CeRNA to modulate miR489-3p/ARD1
Source: Sci Rep. 2025 Aug 26;15:31501. doi: 10.1038/s41598-025-17372-9 (PMC12381120; doi:10.1038/s41598-025-17372-9)
Supplement: Supplementary file 3 — Supplementary Material 3 [file 41598_2025_17372_MOESM3_ESM.docx]

**Supplementary Figure**

Figure S1

(A) ARD1 expression was analyzed in the Breast Cancer Gene-Expression Miner v4.6, with a comparison between the tumor-adjacent and tumor tissues; (B) The disease-free survival (DFS) analysis of breast cancer by microarray data; (C) overall survival (OS) analysis of breast cancer by microarray data; (D) distant metastasis-free survival (DMFS) analysis of breast cancer by microarray data; (E) The DFS analysis of breast cancer by RNA-seq data; (F) overall survival analysis of breast cancer by RNA-seq data; T: tumor tissues; N: adjacent tissues. HR: hazard ratio, ***, p < 0.001.

Figure S2

(A) qRT-PCR verified the knockdown or overexpression efficiency of miR-489–3p in miR-489–3p-mimics or miR-489–3p-inhibitor transfected cells. (B) qRT-PCR was used to detect the effects of miR-489-3p up-regulation and down-regulation on ARD1 mRNA in BC cells.(C) qRT-PCR was used to detect the expression levels of LRRC75A-AS1 in MDA-MB-231 and SK-BR-3 cells after transfection with LRRC75A-AS1 or si-LRRC75A-AS1. (D) The effects of LRRC75A-AS1 overexpression and knockdown on ARD1 mRNA in BC cells were detected by qRT-PCR. *, p < 0.05; **, p < 0.01, ***, p < 0.001.
